# Lésion verruqueuse nasale: un cas marocain de leishmaniose cutanée atypique

**DOI:** 10.48327/mtsi.v5i4.2025.681

**Published:** 2025-10-09

**Authors:** Khalil ZIMI, Imane ZOUAOUI, Hanaa NEJJARI, Sarra AOUFI

**Affiliations:** 1 Laboratoire central de parasitologie-mycologie, Centre hospitalo-universitaire (CHU) Ibn Sina, Rabat, Maroc; 2 Faculté de médecine et de pharmacie de Rabat, Impasse souissi, Rabat 10100, Maroc

**Keywords:** Leishmaniose cutanée nasale, *Leishmania infantum*, PCR-RFLP, Maroc, Afrique du Nord, Nasal Cutaneous Leishmaniasis, *Leishmania infantum*, PCR-RFLP, Morocco, North Africa

## Abstract

**Introduction/Justification:**

La leishmaniose cutanée (LC) est une parasitose endémique au Maroc. Elle est particulièrement préoccupante en raison de la diversité des espèces responsables et de leurs manifestations cliniques variées. *Leishmania infantum,* souvent associé à la leishmaniose viscérale, peut également provoquer des formes cutanées localisées dans des contextes épidémiologiques particuliers. Nous rapportons un cas atypique de LC nasale causée par *L. infantum.*

**Observation:**

Une patiente de 60 ans, résidant à Salé et sans antécédent médical notable, a présenté une lésion inflammatoire verruqueuse sur l’aile gauche du nez, apparue six mois après un séjour dans la ville de Taza. Lexamen clinique et l’analyse parasitologique ont permis de poser le diagnostic de leishmaniose cutanée. L’identification de l’espèce *(L. infantum)* a été réalisée par PCR-RFLP. Une cicatrisation partielle de la lésion a été obtenue après traitement par antimoniate de méglumine.

**Discussion/Conclusion:**

Ce cas rapporte une présentation atypique sous forme de lésion nasale verruqueuse rare de LC à *L. infantum,* espèce classiquement associée à la forme viscérale mais de plus en plus rapportée dans des manifestations cutanées localisées, témoignant ainsi de la diversité clinique de la LC. Le retard diagnostique n’a permis qu’une cicatrisation partielle, soulignant l’importance d’une reconnaissance précoce des formes inhabituelles. Ce cas souligne également la nécessité de renforcer la surveillance épidémiologique et de sensibiliser davantage les professionnels de santé ainsi que les populations exposées, dans le cadre d’une stratégie intégrée de lutte contre la LC.

## Abstract (en)

**Introduction/Justification:**

Cutaneous leishmaniasis (CL) is an endemic parasitic disease in Morocco. It is of particular concern due to the diversity of species involved and their varied clinical manifestations. *Leishmania infantum,* which is often associated with visceral leishmaniasis, can also cause localized cutaneous forms of the disease in certain epidemiological contexts. We present a case of atypical nasal CL caused by *L. infantum.*

**Observation:**

A 60-year-old female patient residing in Salé with no significant medical history presented with a verrucous inflammatory lesion on the left side of her nose. The lesion appeared six months after she stayed in the city of Taza. Clinical examination and parasitological analysis led to a diagnosis of cutaneous leishmaniasis. PCR-RFLP identified the species as *L. infantum.* The lesion partially healed after treatment with meglumine antimoniate.

**Discussion/Conclusion:**

This case reports an atypical presentation of CL in the form of a rare verrucous nasal lesion caused by *L. infantum.* This species is classically associated with visceral leishmaniasis, but it is increasingly being reported in localized cutaneous manifestations. This case demonstrates the clinical diversity of CL. The delay in diagnosis resulted in only partial healing, which highlights the importance of early recognition of unusual forms. This case also underscores the need to strengthen epidemiological surveillance and raise awareness among healthcare professionals and exposed populations as part of an integrated strategy to combat CL.

## Introduction

La leishmaniose cutanée (LC) constitue un problème majeur de santé publique au Maroc. Elle est due à trois espèces du genre *Leishmania*: *L. major, L. tropica* et *L. infantum* [3,5, 7,9, 10,12,13]. Chacune de ces espèces présente un contexte épidémiologique distinct ainsi qu’un polymorphisme clinique spécifique [11]. Dans cet article, nous rapportons un cas sporadique de LC nasale causée par *L. infantum,* caractérisé par un aspect morphologique inhabituel. L’objectif est de mettre en lumière les particularités épidémiologiques et cliniques de cette forme atypique de leishmaniose.

## Description du cas

**Figure 1:**
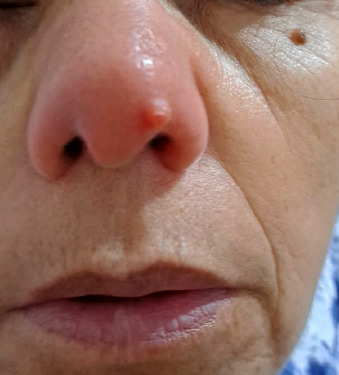
Lésion initiale inflammatoire survenue six mois après la piqûre

**Figure 2:**
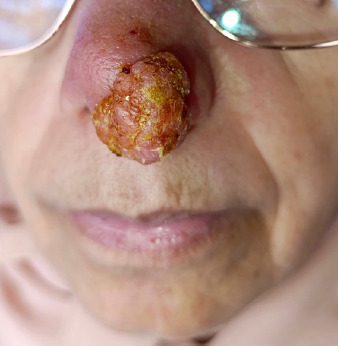
Évolution de la lésion inflammatoire sur l’aile gauche et le lobe du nez après 1 an et 3 mois présentant un aspect verruqueux

Il s’agit d’une patiente de 60 ans, résidant dans la ville de Salé au nord-ouest du Maroc. Elle a été adressée au laboratoire central de parasitologiemycologie pour suspicion d’une leishmaniose cutanée. À l’interrogatoire la patiente a rapporté qu’elle avait ressenti une piqûre d’insecte pendant son séjour à Taza lors d’un voyage. Une petite lésion inflammatoire au niveau nasal est apparue six mois après son séjour (Fig. 1). Elle s’est étendue neuf mois après son apparition sur l’ensemble de l’aile gauche du nez malgré un traitement local par des antibiotiques. L’examen clinique a objectivé une lésion cutanée de type inflammatoire, saillante et d’aspect verruqueux au niveau de l’aile gauche et du lobe du nez (Fig. 2).

Le prélèvement a été réalisé par raclage des bordures des ulcérations. Les échantillons obtenus ont été colorés au Giemsa et examinés au grossissement x 100.

**Figure 3:**
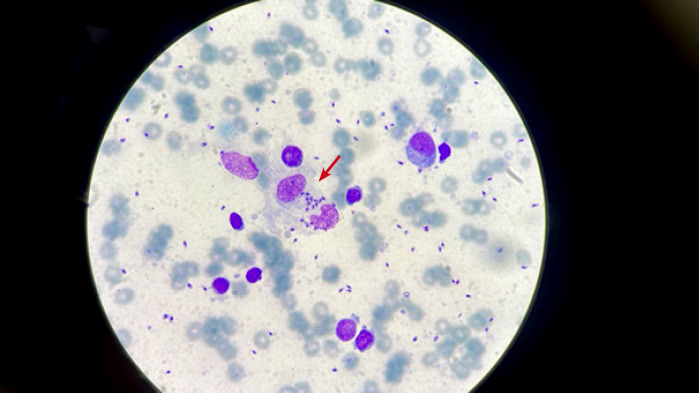
Formes amastigotes de *Leishmania* sp à l’objectif 100. La flèche indique les formes amastigotes de leishmanies

L’observation au microscope optique a montré des formes amastigotes de *Leishmania* sp. au sein et à l’extérieur des macrophages (Fig. 3), confirmant ainsi le diagnostic de LC. L’identification spécifique a été réalisée au Laboratoire national de référence des leishmanioses de l’Institut national d’hygiène, en utilisant la technique de PCR-RFLP. L’analyse du profil électrophorétique a révélé une correspondance avec celui de *L. infantum.*

En parallèle, un examen anatomo-pathologique a été réalisé sur deux fragments biopsiques. Il a montré que le cytoplasme des macrophages était le siège de corps granuleux et basophiles en faveur de corps de Leishman.

Le diagnostic de LC à *L. infantum* a été retenu. Un traitement à base d>antimoniate de méglumine (Glucantime) a été initié, selon le protocole décrit par le ministère de la Santé: 20 mg de Sb5+/kg ont été administrés par voie intramusculaire sans dépasser deux ampoules, pendant trois semaines. L’évolution a été marquée par une cicatrisation partielle de la lésion.

## Discussion

La LC constitue un problème de santé publique au Maroc, en raison de sa large distribution géographique et de la diversité des espèces responsables, notamment *L. tropica, L. major* et *L. infantum* [5,9, 10,13]. Dans notre cas, la patiente résidait dans une région de faible endémie mais avait voyagé dans la région de Taza, située au nord-est du Maroc. Cette région connaît une recrudescence des cas de LC à *L. tropica* [11]. Selon l’étude de Hakkour *et al.* [6], de nouvelles données épidémiologiques mettent en évidence la coexistence de foyers de leishmaniose viscérale (LV) et LC dans la région de Taza. Les zones rurales proches des massifs forestiers, abritant des chiens errants et des phlébotomes, notamment *Phlebotomus perniciosus* et *P. ariasi* proches des habitations, ont été identifiées comme des foyers actifs de transmission.

Classiquement, la LC se manifeste par une papule érythémateuse évoluant vers une ulcération centrale avec des bords surélevés. La forme typique est une lésion unique ou multiple, localisée sur les zones découvertes, évoluant lentement vers une cicatrisation spontanée [4]. Dans notre cas, la patiente a présenté une lésion atypique sur le nez, caractérisée par une évolution prolongée et un aspect verruqueux. Ce type de présentation, bien que rare, a été rapporté dans des études antérieures soulignant des formes inhabituelles de LC, incluant des manifestations érysipéloïdes, lupoïdes et angiolupoïdes [5,6, 7,9, 10,12,13]. Ces formes rares s’expliqueraient par des interactions complexes entre le parasite et la réponse immunitaire de l’hôte, laquelle est fortement médiée par l’immunité cellulaire. La réponse immunitaire peut être modulée par divers facteurs, notamment l’âge, l’état nutritionnel, l’utilisation de stéroïdes ou des comorbidités sous-jacentes [2].

Bien que *L. infantum* soit principalement associée à la LV dans plusieurs régions du Maroc, elle est de plus en plus impliquée dans des formes cutanées localisées, notamment dans certains foyers émergents, où les taux d’infection sont particulièrement élevés, confirmant son rôle dans ces manifestations cutanées [3]. Des études récentes, notamment celles menées dans la région de Taza, ont mis en évidence une recrudescence des cas de LC à *L. infantum*, confirmant son rôle dans la diversité clinique de la maladie [6]. Ainsi une série de 40 cas suivie dans le service de dermatologie du CHU Hassan II de Fès entre janvier 2010 et décembre 2015 a montré une variété de formes cliniques, y compris 9 cas en formes verruqueuses et lupoïdes [1]. Ces études confirment non seulement la présence active de foyers mixtes (LC et LV) mais aussi l’implication croissante de *L. infantum* dans les tableaux cutanés atypiques. Dans les pays voisins comme la Tunisie et l’Algérie, les formes cliniques atypiques de la LC, notamment les lésions verruqueuses, sont bien décrites et relativement fréquentes. En Tunisie, des études menées dans le centre et le sud du pays ont mis en évidence une proportion significative de présentations verruqueuses, parfois dépassant 25% des cas. De même en Algérie, plusieurs publications font état d’un polymorphisme clinique important, incluant des formes verruqueuses, lupoïdes ou nodulaires [4,5, 6,7, 8,9]. En revanche, au Maroc, ces formes restent rares et peu rapportées dans la littérature.

Le traitement initié à base d’antimoniate de méglumine a suivi les recommandations nationales, bien que le retard de diagnostic ait compromis une résolution complète des lésions [4]. Une cicatrisation partielle a été observée, soulignant l’importance de la détection précoce et d’un traitement rapide pour limiter les séquelles.

## Conclusion

Ce cas illustre la complexité et le polymorphisme clinique de la leishmaniose cutanée, en particulier lorsqu’elle est causée par *L. infantum,* une espèce habituellement associée aux formes viscérales. La présentation atypique, caractérisée par une lésion verruqueuse, met en évidence l’importance d’une reconnaissance précoce des formes rares afin d’éviter les retards diagnostiques et thérapeutiques.

## Consentement de la patiente

Le consentement écrit de la patiente a été obtenu.

## Financement

Cette étude n’a bénéficié d’aucun financement.

## Contributions des auteurs et autrices

Khalil ZIMI: conception de l’étude, prospection bibliographique, définition de la méthodologie, interprétation des résultats et rédaction du manuscrit.

Imane ZOUAOUI: supervision de l’étude, confirmation de l’exactitude et de l’intégrité des données, approbation et validation de la version finale Hanaa NEJJARI: conception de l’étude, prospection bibliographique et rédaction du manuscrit Sarra AOUFI: approbation de la version finale

## Déclaration de liens d’intérêt

Aucun lien d’intérêt n’a été déclaré.
