# Supplementary material for: Nasal wart lesion: a Moroccan case of atypical cutaneous leishmaniasis
Source: Med Trop Sante Int. 2025 Oct 9;5(4):mtsi.v5i4.2025.681. [Article in French] doi: 10.48327/mtsi.v5i4.2025.681 (PMC12948355; doi:10.48327/mtsi.v5i4.2025.681)
Supplement: Related texts

# Research article: Nasal wart lesion: a Moroccan case of atypical cutaneous leishmaniasis

## Introduction

Cutaneous leishmaniasis (CL) is a significant public health concern in Morocco. It is caused by three species of the genus *Leishmania: L. major, L. tropica,* and *L. infantum* [3,5,7,9,10,12,13]. Each species has a distinct epidemiological context and specific clinical manifestations [11]. This article reports a sporadic case of nasal CL caused by *L. infantum* that was characterized by an unusual morphological appearance. Our goal is to highlight the epidemiological and clinical features of this atypical form of leishmaniasis.

## Case description

The patient is a 60-year-old woman who resides in Salé, a city in northwestern Morocco. She was referred to the central parasitology-mycology laboratory for suspected cutaneous leishmaniasis. During the interview, she reported feeling an insect bite while traveling in Taza. Six months after her trip, a small inflammatory lesion appeared on her nose (Fig. 1). Nine months after its appearance, despite local treatment with antibiotics, it had spread across the entire left side of her nose. Clinical examination revealed an inflammatory, protruding, wart-like skin lesion on the left side of her nose and nasal lobe (Fig. 2).

A sample was obtained by scraping the edges of the ulcerations. The samples were stained with Giemsa and examined at 100x magnification. Observation under an optical microscope revealed amastigote forms of *Leishmania* sp. within and outside macrophages (Fig. 3), confirming the diagnosis of CL. The National Reference Laboratory for Leishmaniasis at the National Institute of Hygiene performed specific identification using the PCR-RFLP technique. Analysis of the electrophoretic profile revealed a match with *L. infantum*.

At the same time, a pathological examination was performed on two biopsy fragments. The examination showed that the macrophages’ cytoplasm contained granular and basophilic bodies consistent with Leishman bodies.

The diagnosis of CL caused by *L. infantum* was confirmed. According to the protocol described by the Ministry of Health, treatment with meglumine antimoniate (Glucantime^®^) was initiated: 20 mg of Sb5+/kg was administered intramuscularly over three weeks, not exceeding two ampules. The outcome was partial healing of the lesion.

**Figure 1:** Initial inflammatory lesion occurring six months after the bite

**Figure 2:** Progression of the inflammatory lesion on the left wing and lobe of the nose after 1 year and 3 months, presenting a wart-like appearance

**Figure 3:** Amastigote forms of *Leishmania* sp at 100x magnification. The arrow indicates the amastigote forms of Leishmania

## Discussion

CL is a public health problem in Morocco due to its wide geographical distribution and the diversity of species involved, particularly *L. tropica, L. major,* and *L. infantum* [5,9,10,13]. In this case, the patient lived in an area of low endemicity but traveled to the Taza region in north-eastern Morocco. This region has experienced a resurgence of CL cases caused by *L. tropica* [11]. According to Hakkour *et al’s* study [6], new epidemiological data highlight the coexistence of visceral leishmaniasis (VL) and CL foci in the Taza region. Active transmission foci have been identified in rural areas close to forests, where stray dogs and sand flies, particularly *Phlebotomus perniciosus* and *P. ariasi,* are present near human dwellings.

CL typically manifests as an erythematous papule that progresses to central ulceration with raised edges. It usually presents as one or more lesions on exposed areas that slowly progress to spontaneous healing [4]. In this case, however, the patient presented with an atypical lesion on the nose that was characterized by a prolonged course and verrucous appearance. Although rare, this type of presentation has been reported in previous studies highlighting unusual forms of CL, including erysipeloid, lupoid, and angiolupoid manifestations [5,6,7,9,10,12,13]. These rare forms can be explained by the complex interactions between the parasite and the host’s immune response, which is strongly mediated by cellular immunity. This response can be influenced by various factors, such as age, nutritional status, steroid use, and underlying comorbidities [2].

*Leishmania infantum* is primarily associated with VL in Morocco; however, it is increasingly implicated in localized cutaneous forms, particularly in emerging regions with high infection rates, confirming its role in these manifestations [3]. Recent studies, particularly those conducted in the Taza region, have highlighted an increase in CL cases caused by *L. infantum,* confirming its role in the disease’s clinical diversity [6]. A series of 40 cases observed in the dermatology department at Hassan II University Hospital in Fez from January 2010 to December 2015 demonstrated various clinical presentations, including nine cases of verrucous and lupoid forms [1]. These studies confirm the active presence of mixed foci (CL and VL) and the growing involvement of *L. infantum* in atypical skin presentations.

In neighboring countries such as Tunisia and Algeria, atypical clinical forms of CL, particularly verrucous lesions, have been well documented and are relatively common. Studies conducted in central and southern Tunisia have revealed a substantial proportion of verrucous presentations, sometimes exceeding 25% of cases. Similarly, several publications from Algeria report significant clinical polymorphism, including verrucous, lupoid, and nodular forms [4,5,6,7,8,9]. In Morocco, however, these forms are rare and rarely reported in the literature.

Treatment with meglumine antimoniate was initiated in accordance with national recommendations; however, the delay in diagnosis compromised complete resolution of the lesions [4]. Partial healing was observed, underscoring the importance of early detection and prompt treatment to minimize sequelae.

## Conclusion

This case illustrates the complexity and clinical polymorphism of cutaneous leishmaniasis, particularly when caused by *L. infantum,* a species typically linked to visceral forms. This unusual presentation, characterised by a wart-like lesion, highlights the importance of early recognising rare forms on, to avoid delays in diagnosis and treatment.

## Patient’s consent

The patient’s written consent was obtained.

## Funding

This study did not receive any funding.

## Authors’ contributions

Khalil ZIMI: study design, literature review, definition of methodology, interpretation of results, and manuscript writing.

Imane ZOUAOUI: study supervision, confirmation of data accuracy and integrity, approval and validation of the final version Hanaa NEJJARI: study design, literature review, and manuscript writing Sarra AOUFI: approval of the final version

## Conflict of interest

The authors declare no conflict of interest.
